# AC/DC Thermal Nano-Analyzer Compatible with Bulk Liquid Measurements

**DOI:** 10.3390/nano12213799

**Published:** 2022-10-28

**Authors:** Yaroslav Odarchenko, Anna Kaźmierczak-Bałata, Jerzy Bodzenta, Enrico Ferrari, Mikhail Soloviev

**Affiliations:** 1 Department of Biological Sciences, Royal Holloway University of London, Egham, Surrey TW20 0EX, UK; 2 Institute of Physics, Center for Science and Education, Silesian University of Technology, Konarskiego 22B, 44-100 Gliwice, Poland; 3 Department of Life Sciences, University of Lincoln, Lincoln LN6 7TS, UK

**Keywords:** localized sensing, thermal analysis, AC nanocalorimetry, applied biophysics

## Abstract

Nanocalorimetry, or thermal nano-analysis, is a powerful tool for fast thermal processing and thermodynamic analysis of materials at the nanoscale. Despite multiple reports of successful applications in the material sciences to study phase transitions in metals and polymers, thermodynamic analysis of biological systems in their natural microenvironment has not been achieved yet. Simply scaling down traditional calorimetric techniques, although beneficial for material sciences, is not always appropriate for biological objects, which cannot be removed out of their native biological environment or be miniaturized to suit instrument limitations. Thermal analysis at micro- or nano-scale immersed in bulk liquid media has not yet been possible. Here, we report an AC/DC modulated thermal nano-analyzer capable of detecting nanogram quantities of material in bulk liquids. The detection principle used in our custom-build instrument utilizes localized heat waves, which under certain conditions confine the measurement area to the surface layer of the sample in the close vicinity of the sensing element. To illustrate the sensitivity and quantitative capabilities of the instrument we used model materials with detectable phase transitions. Here, we report ca. 10^6^ improvement in the thermal analysis sensitivity over a traditional DSC instrument. Interestingly, fundamental thermal properties of the material can be determined independently from heat flow in DC (direct current) mode, by using the AC (alternating current) component of the modulated heat in AC/DC mode. The thermal high-frequency AC modulation mode might be especially useful for investigating thermal transitions on the surface of material, because of the ability to control the depth of penetration of AC-modulated heat and hence the depth of thermal sensing. The high-frequency AC mode might potentially expand the range of applications to the surface analysis of bulk materials or liquid-solid interfaces.

## 1. Introduction

**Figure 1:**
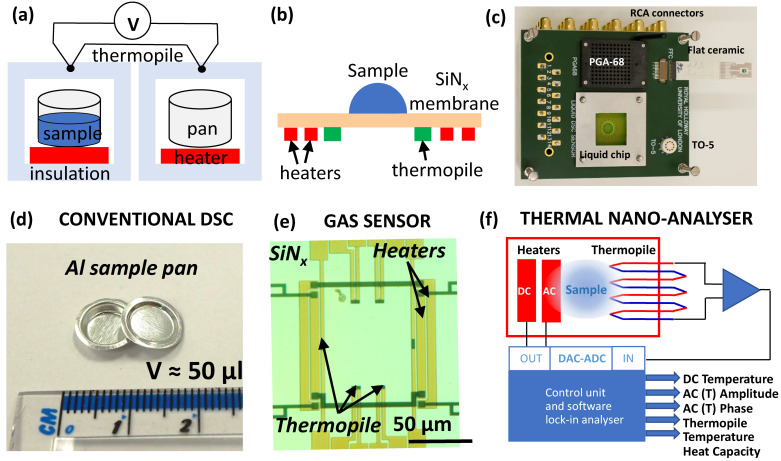
Thermal analysis using conventional differential scanning calorimetry (DSC) and thermal nano-analyzer. Schematics of (**a**) DSC instrument with two macroscopic pans for sample and reference; (**b**) sensor with two uninsulated heaters and thermopile placed on a 1 µm thick SiNx membrane, the sample may be in ambient atmosphere or in liquid, whilst non-conducting liquids are compatible with both sides of the sensor membrane, (**c**) sensor adapter compatible with 15 nL thermo-responsive liquid chip as well as gas sensor in TO-5, PGA-68 and flat ceramic housing, (**d**) commercial DSC aluminium pan and lid requiring 50 µL sample volume, (**e**) gas sensor active area of 100 × 100 µm^2^ with 2 pairs of heaters and 8 spot thermopile, (**f**) schematics of thermal nano-analyzer reported in this work.

The science of calorimetry covers multiple thermo-analytical techniques, which usually measure temperature and/or heat flow due to chemical or physical transitions in the sample analyzed, the latter typically induced by changing the sample temperature. Differential thermal analysis (DTA) and differential scanning calorimetry (DSC) measure the difference in either temperature or heat flux between sample and reference undergoing identical temperature (in DSC) or heating (in DTA) modes. DSC and DTA offer high sensitivity and are widely used, especially in materials sciences, for studying metals, alloys, ceramics, polymers and other materials, including macromolecules and organic/inorganic low-molecular-weight compounds and life sciences for studying solid-state pharmaceutical materials. DSC has been also applied for studying protein stability, protein folding and unfolding, protein phase transitions and interactions, as well as in protein crystallization research [1]. Commercially available DSC instruments are largely limited to the analysis of small dry samples (Figure 1), which limits the range of their applications. Another thermal analytic technique, which does not have such a limitation and is more widely used with liquid samples, is isothermal titration calorimetry (ITC). ITC is especially suitable for in vitro measurements of molecular interactions such as binding affinities and stoichiometry, as well as the analysis of binding thermodynamics. Life science applications of ITC typically involve measuring enthalpy and entropy changes associated with protein–protein, protein-nucleic acid, protein-ligand, protein conformational changes and other inter- and intra-molecular interactions. Whilst ITC is suitable for a liquid water-based sample environment, it is largely limited to in vitro titration measurements, requires high concentration of molecular species and simplified model experimental reaction conditions. ITC relies on a theoretical model to fit the experimental thermograms to extract the results and is relatively slow as a complete titration experiment may last many hours [2].

The ability to determine thermodynamic parameters that underpin materials’ properties and other phase transformations firmly established calorimetry as the “gold standard” for characterizing molecular interactions in material and life sciences. However, despite the widespread and growing use of the calorimetry analysis the commercial instruments typically require relatively high quantities and concentrations of materials and are still severely limited in their ability to use micro- and nano-scale samples, e.g., a typical sample load volume for ITC is 300 µL and 5–50 µL for DSC or to perform spatially resolved 2D or 3D thermal imaging. Combining an integrated thermal probe (thermocouple or resistive) with the Surface Force Microscopy (SFM) principle and Atomic Force Microscopy (AFM) tip in a Scanning thermal microscopy (SThM) approach achieves good lateral resolution of surface temperature measurements in scanning mode (reviewed in [3]). Early SThM efforts achieved a spatial resolution of the order of ~100 nm [4], which was further improved by using active resistive probes, acting both as a heat source and a thermometer [5]. Thermal transitions recorded by SThM in the micrometre vicinity of such a probe match glass transitions and melting and re-crystallizations temperatures for bulk amorphous and crystalline polymers [5]. Subsequently SThM was applied to study a variety of materials under different scanning conditions (reviewed in [6]). The development of SThM further led to the concept of sub-surface scanning thermal microscopy, capable of detecting local differences in density, heat capacity or thermal conductivity below the sample surface. One of the first examples of such sub-surface SThM with lateral resolution of around a micron and depth measurements of a few microns, was thermal conductivity measurement in zero frequency mode (DC only) by using a Wollaston wire with a platinum tip, although only relatively large differences in thermal conductivities were detectable (micron size metallic particles were embedded in a polymer matrix) [7]. The experimental heat flow and temperature profiles as a function of location depth and lateral distance between the particles has been modelled with a one-dimensional theoretical model [7]. Further details are provided in the section “Dynamic heat propagation”.

Alternative approaches for sub-surface evaluation of materials are provided by infrared (IR) thermography or ultrasound AFM. IR thermography is a non-destructive thermo-analytic method for contact or non-contact near-surface inspection of solid materials. Surface or sub-surface features or defects affect heat conduction thus resulting in the temperature gradient that can be conveniently detected by an IR camera. Lock-in thermography is capable of revealing sub-surface defects or other material irregularities by measuring a phase shift between optical AC heating and the surface thermal emission or temperature [8]. In a modification of the original principles, the heating might be provided by modulated ultrasound, which would also result in selective heating of material defects [9]. Other distantly related techniques of ultrasound AFM (UFM) [10,11], atomic force acoustic microscopy (AFAM) [12], ultrasonic atomic force microscopy (UAFM) [13], and the related Scanning Near-Field Ultrasound Holography (SNFUH), alternatively called Heterodyne Force Microscopy (HFM), are also capable of high resolution imaging of subsurface features, defects and elastic but not thermal properties, with a good spatial resolution of the order of 10–100 nm [14] or for imaging biological materials [15]. These methods and later derivatives, similar to the lock-in thermography, rely largely on amplitude and/or phase sensitive detection to reveal sub-surface features. Other relevant developments are linked to the adaptation of the 3-omega (3*ω*) technique for thermal analysis of bulk materials, including biological materials. For example, insulated and supported thin wire 3*ω* sensors have been used to study thin and bulk biological materials [16]. If the thermal diffusion length is less than the sample material thickness, the local thermal ‘waves’ generated with AC heating are confined within the material boundaries. Under such conditions and following calibration of the empty sensors to compensate for the wire support material, this approach may be used to measure thermal conductivity of the materials in contact with the 3*ω* sensor [17]. One other interesting feature of the 3*ω* sensing approach is the ability to utilize the frequency dependence of thermal waves propagation which provides new opportunities, e.g., detection of nearby phase boundaries [18]. Notwithstanding the limited sensitivity of resistive sensors and other limitations of the supported 3*ω* electrothermal techniques, such measurement approach may be realized in the form of thermal sensors integrated with traditional medical instruments [19]. Other recent developments have provided a variety of miniaturized titration calorimeters [20] and other microfluidic based instruments, and together with ingenious experimental approaches suitable for the analysis of fast reactions [21] proteins in solution [22] or isolated single cells [23]. Nevertheless, despite tremendous recent advances in nanotechnology, smart materials, and biological sciences, including molecular and drug screening, traditional calorimetric techniques retain some of their limitations, such as inability to measure thermal properties of nanosized quantities of materials in bulk liquids or to characterize biomolecular interactions in vivo.

One of the solutions to tackle challenges associated with measuring thermal properties of nanostructured materials or fast processes is chip-based calorimetry which emerged in the 1990s. The first reports, published almost simultaneously by L. Allen et al. [24] and D.W. Denlinger et al. [25] described microcalorimeters based on thin films generated with semiconductor processing technology. Nanocalorimetry detection allows the study of nanogram quantities of sample at high heating/cooling rates (up to 10^6^ °C·s^−1^), thus providing an advantage over conventional instruments [26,27], that are normally capable of scanning rates up to several °C·s^−1^ only.

Subsequent publications brought about impressive advances in the fields of isothermal microcalorimetry [28] and fast scanning DSC [29] including early commercial developments [30,31]. A growing number of published studies report applications of fast thermal analyzes to micro- and nano-sized samples. These largely fall into two major categories—open chamber sensors for use with micro- or nano-sized solid materials or liquid droplets [32], e.g., for the analysis of proteins [33,34], or miniaturized enclosed micro-fluidic chambers [35,36,37], some within nanolitre range [38] or even as small as few picolitres [39].

A common feature of the vast majority of recent studies is physical miniaturization of the samples. However, the possibility of being able to probe samples in bulk liquid media, rather than miniaturized liquid cells, has not been reported to the best of our knowledge. Such capability would be particularly important for life science applications, where it is not always possible to reduce the physical size of biological objects to suit the nano-sized sensors, or to take biological objects from their native bulk liquid environment. The majority of applications of fast calorimetry remain in material sciences rather than life sciences. Most recent examples include studies of fast heating and cooling rates, of melt memory effects, polymer crystallization and glass formation [40,41,42] material aging [43] or tribology [44]. Another recent trend in this area is the integration of chip calorimetry with other instrumental analyzes such as X-ray diffraction [45].

Only a handful of dedicated in vivo studies have been published, mostly employing macroscopic preparations of bacterial cultures [46], with the notable exceptions of the direct real-time calorimetry study of an individual *C. elegans* worm [47] or unicellular *Paramecium caudatum* [48]. Some further advances were achieved with commercially available instruments such as Flash DSC [34,49,50] or TA Instruments [37] largely used to study lysozyme protein—an old favourite model protein continuously explored over the last 50 years. However, despite the consistent tendency towards miniaturization, simply scaling down traditional calorimetric techniques, although beneficial for material sciences, may not work well for biological objects, which cannot always be scaled down to suit instrument size or taken out of their environment. To the best of our knowledge, no studies achieved thermodynamic analyzes of biomolecules in their localized natural environment either in vitro or in vivo.

### Dynamic Heat Propagation

In the simplest case of 1D heat transport inside a solid material, the Fourier’s differential equation can be written as

(1) $$\frac{\partial T}{\partial t}=\alpha \frac{\partial^{2}T}{\partial x^{2}}\;$$

where *T* is temperature, *t* is time, and *α* is thermal diffusivity. Let us assume that the semi-infinite solid occupying the half space *x* < 0 is heated by a harmonic heat flux of the density

(2) $$-K\frac{\partial T}{\partial x}|\begin{array}{c} x=0 \end{array}=-\frac{1}{2}I_{0}\left(1-\cos\omega t\right),$$

where *K* is a thermal conductivity, *I*_0_ is the maximum of heat flux density, and *ω* is the angular frequency of the AC heat flux component. The solution of Equation (1) for temperature disturbance *θ* = *T* − *T*_DC_, with the boundary condition (2), is given in Equation (3):

(3) $$\theta =T_{0}e^{-\frac{x}{\mu }}\cos\left(\omega t-\frac{x}{\mu }-\frac{\pi }{4}\right)\;$$

where *T*_DC_ is the DC temperature component, and *µ* is thermal diffusion length [51], which can be defined as described in Equation (4):

(4) $$\mu =\sqrt{\frac{2\alpha }{\omega }}=\sqrt{\frac{K}{\pi c_{p}\rho f}}$$

where *K* is thermal conductivity, *c*_p_ is specific heat, *ρ* is density and *f = ω*/2π stands for frequency of the AC heat flux component.

Features embedded at a depth inside a material should be detectable if the thermal diffusion length *µ* is comparable to or larger than the detectable feature size and its depth. For example, assuming a thermal modulation frequency of 50 Hz, the thermal diffusion length in a polymer such as polypropylene with thermal diffusivity of 9.6·10^−4^ m^2^ s^−1^ is estimated to be 25 µm. The same calculation for the SiN_x_ with a thermal diffusivity of 1.07·10^−5^ m^2^ s^−1^ is estimated to be 260 µm.

In this paper, we aim to show that an AC-modulated thermal analyzer instrument is capable of quantitative sensing of ‘ng’ level quantities of material under ambient conditions (air) or immersed in bulk liquid. We chose to test the instrument using well characterized model materials with detectable phase transitions, such as Indium and Polyethylene glycol (PEG), to better illustrate the quantitative capabilities of the instrument and to facilitate comparisons. Our results demonstrate superiority of our thermal nano-analyzer over conventional DSC in terms of sensitivity, the ability to work at very fast as well as slow heating rates (comparable to traditional DSC) and the ability to limit the sensing depth by adjusting AC modulation. Localized sensing enables the analysis of ‘ng’ level quantities of materials in bulk liquid environments, which potentially offers new opportunities for studying thermodynamics of biological objects, e.g., individual cells, cell compartments or individual organelles and, ultimately individual biopolymer molecules, such as enzymes, without removing them from their macroscopic environment and without using artificially introduced markers or inserting any sensors into such cells or organelles.

## 2. Materials and Methods

### 2.1. Materials

Indium (Sigma-Aldrich, purity 99.99%, Sigma-Aldrich, St. Louis, MI, USA) was used as calibration material with known temperature and enthalpy of melting [52] Immersol 518 F (Carl Zeiss Ltd., Cambridge, UK) oil was used as electrically nonconductive liquid environment. Polyethylene glycol (PEG, M_w_ = 8000 M) solution in water (40% *w*/*w*) from Sigma-Aldrich and was used without any dilution. This polymer, with defined chemical composition, has a melting temperature in the physiological range (25–95 °C) and was chosen as model material to test the performance of the new testing platform.

### 2.2. Differential Scanning Calorimetry (DSC)

Conventional DSC analysis was conducted using a Perkin Elmer Diamond DSC. The scanning rates were 2, 5, 10, 20, 50, 200 and 500 °C min^−1^. A heating rate of 500 °C min^−1^ was the maximum scanning rate achievable with the commercial instrument. Dried PEG film was obtained by drying polymer solution and sealed in an aluminium pan of 50 μL volume (Figure 1d). The typical sample weight was between 5 and 20 mg. The DSC instrument was calibrated using melting of indium.

### 2.3. Thermal Nano-Analyzer

A simplified block diagram of our custom-built thermal nano-analyzer, and an image of our multi-sensor holder are shown in Figure 1c,e,f. The new instrument design was adapted from the earlier reported [53], but the instrument has much expanded sensor compatibility, including with all of the existing XEN-39390 series sensors in TO-5, PGA-68 and flat ceramic housing (Supplementary Figure S1). The new instrument is also suitable for use with liquid cells, such as XEN-39400liq sensors (Xensor Integration, Delfgauw, Netherlands) and allows the use of other external sensors via adaptors. The resistive heaters on the nano-analyzer sensor chip are controlled through a programmable 16-bit, 1 MHz ADC-DAC board DaqBoard/3000USB (Measurement Computing Corporation, Norton, MA, USA). All electric heating parameters are fully software-controlled (Supplementary Figure S1). The instrument is configured to allow two independent resistive heaters to be employed simultaneously but controlled separately.

The sensor active area with the integrated resistive heaters and thermopiles is illustrated in Figure 1e. Linear (DC) and modulation (AC) modes were typically employed. In DC mode short linear voltage pulses are applied to one of the resistive heaters. This mode allows slow and fast scanning rates up to 150,000 °C min^−1^. In AC mode a modulation current is applied generating calibrated AC heat on top of any linear heating/cooling rate, or isothermally. Change in amplitude and phase of temperature modulation due to the heat flow from the sample can be conveniently detected at scanning rates from 2 to 1000 °C min^−1^.

Measurements reported here were conducted using XEN-39392 (Xensor Integration, Netherlands) and a custom-made liquid cell adaptor. Nanogram weight PEG particles were cut out from a PEG film made from the polymer solution and deposited on the thin suspended SiN_x_ membrane of the sensor. The thermograms measured at different heating rates have been corrected for the time delays associated with each heating rate. Indium microparticles were used for temperature calibration and to analyze the quantitative nature and linearity of sensor responses.

In AC mode the amplitude of temperature modulation of the sample can be described by Equation (5):

(5) $$T_{mod}=\frac{P}{\sqrt{m^{2}c^{2}\omega^{2}+Q^{2}}}\;$$

where *P* is amplitude of modulating power, *m* is mass of the sample, *c* is specific heat, *ω* denotes angular modulation frequency and *Q* is the heat transfer coefficient between the sensor and the environment [54]. The heat flow in AC mode was calculated using the formula derived for TMDSC [55]:

(6) $$\frac{dQ\left(\mathrm{AC}\right)}{dt}=mcT_{mod}\omega \;$$

where *Q*(AC) is the heat generated during AC modulation.

### 2.4. Modelling of the Heat Propagation

The heat transfer module in COMSOL Multiphysics was used to model AC heat propagation through the sensor and its immediate surroundings as described previously [30,31]. The geometry of the electro-thermal finite element model FEM of the sensor-air system, built in COMSOL Multiphysics. Only half of the sensor was modelled due to the symmetry of the system.

The boundary and initial conditions set into the model comprised the initial temperature of 293.15 K and electric potential of 0 V. Simulations were carried out for both AC heat source with instant power

(7) $$P\left(t\right)=\frac{1}{2}P_{0}\left(1-\cos\omega t\right)$$

where *P*_0_ is AC peak power; and transient process taking place after switching on the DC heat source. The radial and z-direction temperature distributions in the sample were calculated on a regular spatial grid for 0 ≤ r ≤ 500 μm, and 0 ≤ z ≤ 200 μm.

## 3. Results

**Figure 2:**
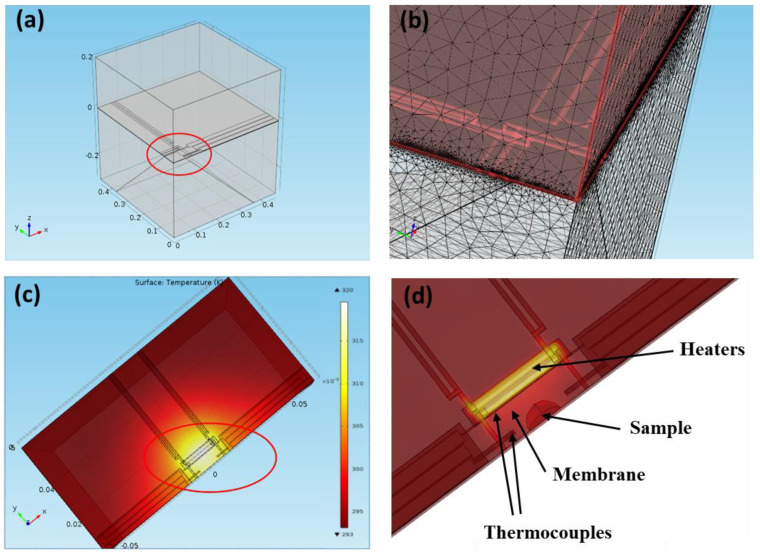
Modelling of the temperature distribution during AC heating for the gas sensor. (**a**) Geometry of the gas sensor with 100 × 100 µm^2^ active area; (**b**) FEM net built using Comsol^TM^; (**c**) Modelling of the temperature distribution during AC heating using two resistive heaters connected in series; (**d**) zoomed-in sensor area with a sample.

We constructed a thermal AC-modulated nano-analyzer suitable for use with a wide range of existing chip-based sensors, having at least one (or a few) resistive heaters and one (or a few) thermocouple-based thermometers (Figure 1e). The instrument relies on AC thermal modulation of the sample and uses software-realized lock-in detection to extract AC thermal response of the sample. A thermopile sensor provides both DC and AC components which are further separated by the instrument hardware and are analyzed separately. The DC component corresponds to the constant temperature of the sensor. The AC readout separates modulated temperature component from the real sensor temperature and allows independent detection of changes in the heat capacity. Unlike traditional DSC heat capacity analysis, the AC sensing under certain conditions will be limited to the vicinity of the sensor, irrespective of the overall size of the sample load. Modelling of the temperature distribution during AC heating confirms that thermal oscillations are localized and limited to the active area of the sensor (Figure 2).

Within the frequency range used with the model (1–100 Hz) the temperature oscillations were localized to the sensing area (Figure 2c,d). Modelling of the temperature distribution during AC heating confirms that thermal oscillations are localized and limited to the active area of the sensor (Figure 2). These results also indicate that under the experimental conditions a sensor with two heaters, such as XEN-39392 can be modelled as a single heat source (Figure 2c). Additionally, our model can describe and quantify heat capacity and thermal conductivity of the system, using the precisely defined geometry of the sensor and thermodynamic properties of the materials used.

**Figure 3:**
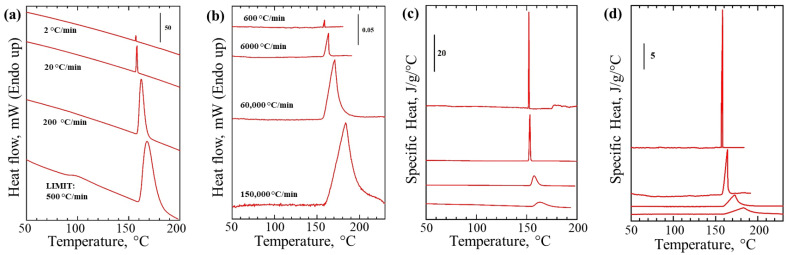
Up to ×100 fold faster scanning rates achieved using thermal nano-analyzer. Melting of indium using DSC and thermal nano-analyzer instruments. Heat flow vs. sample temperature recorded with the help of conventional DSC with 5.76 mg In sample (**a**) and thermal nano-analyzer instrument using 42 ng In particle deposited on the gas sensor (**b**). Heating rates used for each curve are indicated on the graphs. the maximum scanning rate achievable by commercial instrument was 500 °C/min. Two orders of magnitude increase in scanning rate and sensitivity are achieved with the thermal nano-analyzer instrument. Specific Heat vs. sample temperature for DSC (**c**) and thermal nano-analyzer (**d**) were calculated by correcting for heating rate and sample weight.

Proof of principle experiments for new thermal nano-analyzer platform were performed using Indium, traditionally used as DSC calibration standard. Thermograms for Indium recorded using commercial DSC (Figure 3a) and thermal nano-analyzer (Figure 3b) instruments yielded similar endothermic melting peaks in the region 150–180 °C. However, the maximum scanning rate of 150,000 °C min^−1^ used for our thermal nano-analyzer was 300 times faster in comparison to the maximum rate achievable by the conventional DSC instrument used in this study (Figure 3).

Such increased scanning rates are possible due to the much-reduced thermal inertia of the sensor with the sample in our thermal analyzer in comparison with the DSC. The typical DSC sample pan has a volume of 50 μL (Figure 1d) that is equivalent to ca. 50 mg of sample (depending on the material density). Taking the sample mass used in the thermal nano-analyzer to be 1000 ng (higher limit), this corresponds to a decrease in sample mass by 5 × 10^4^. This estimated value is in good agreement with a 300 times gain in thermal inertia, i.e., scanning rate obtained experimentally considering heat loss due to parasitic thermal links and mass of SiNx membrane (Figure 1e). That together with the enhanced signal-to-noise ratio of the instrument input circuits allows a significant improvement in the sensitivity of the new instrument compared to traditional DSC. For example, decrease in sample weight from 5.76 mg in case of DSC down to 42 ng used with the thermal nano-analyzer have not affected measurements of the detected heat of fusion (Figure 3c,d).

**Figure 4:**
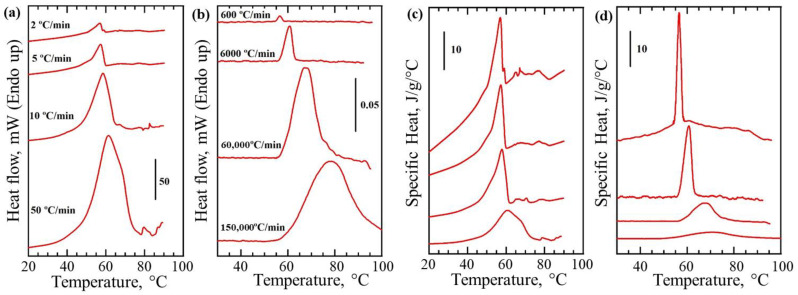
Thermal analysis of model polymer with the melting point closer to physiological temperature range using DSC and thermal nano-analyzer. Heat flow vs. sample temperature recorded with conventional DSC using 19 mg PEG (**a**) and thermal nano-analyzer instrument using 16 ng PEG particle deposited on the sensor (**b**). Heating rates used are shown next to relevant graphs. Artefacts on the DSC curves in the high temperature region are due to the sample leaking. PEG melting can be detected in a wide range of the scanning rates from 600 to 150,000 °C min^−1^. Specific Heat vs. sample temperature for DSC (**c**) and thermal nano-analyzer (**d**) were calculated by correcting for heating rate and sample weight.

Melting of metals occurs at fixed temperatures and therefore could be relatively easily detected by measuring heat flow using calorimetry. Unlike metals, synthetic- and bio-polymers often exhibit heterogeneous crystallization resulting in formation of crystals with different sizes and quality which in turn broadens the recorded melting peak or causes the appearance of multiple endothermic signals on DSC thermograms. To validate the performance of our instrument with materials other than model metals, we used a model polymer PEG, where melting temperature is close to physiological temperature range. Thermograms were recorded at various scanning rates for PEG using both the DSC instrument and our thermal nano-analyzer without using AC modulation (Figure 4a,b). Identically sized PEG samples were used within each series of measurements for each of the instruments. A detected endothermic peak with onset at 55 °C corresponds to the melting of PEG. Excess in heat flow plotted as a function of temperature is also similar for both instruments (Figure 3a,b), however thermal nano-analyzer provided a large gain in sensitivity (note that sample load was only 16 ng which is six orders of magnitude smaller than the one used for the commercial DSC instrument (Figure 4). The endothermic peaks detected for the intrinsically polydisperse crystalline polymer are broader compared with these above obtained for Indium using the same heating rate. The DSC thermogram generated at the scanning rate of 100 °C min^−1^ for PEG sample contains a shoulder at around 70 °C, which becomes more prominent at the higher scanning rates (Figure 4a–c).

There exist two possible explanations for the observed effect. Either there exist two populations of crystals within the sample pan with different melting temperature or leaking of the sample. The existence of different crystal populations can be ruled out from the thermal nano-analyzer data that show single melting peak for all scanning rates (Figure 4b,d). Therefore, possible misinterpretation of the data due to the instrumental artefacts can be avoided with the thermal nano-analyzer. The sensor used for the thermal nano-analyzer requires loading of a very small amount of sample that stays within the active area between the heaters (Figure 1e). Such geometry and scale help avoid sample leaking.

**Figure 5:**
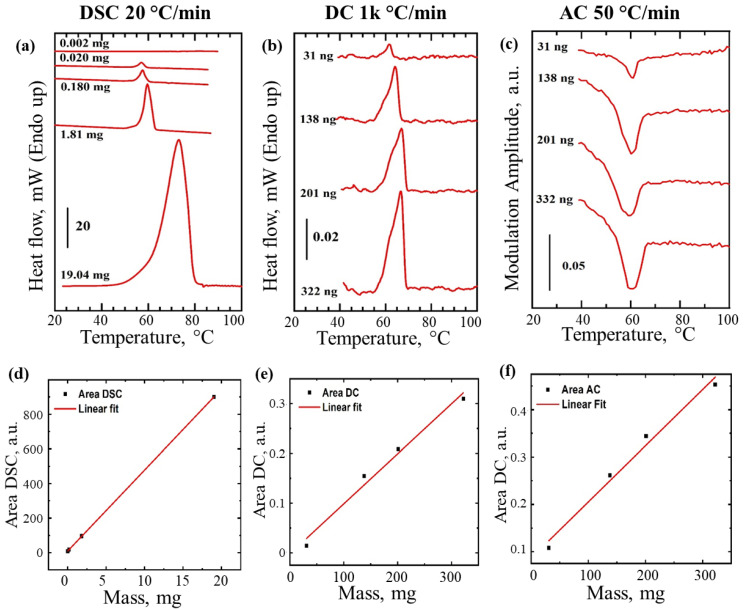
Analysis of polyethylene glycol (PEG) in DC and AC modes. Heat flow vs. sample temperature recorded using traditional DSC instrument (**a**) and thermal nano-analyzer instrument in DC mode (**b**). Heat modulation AC amplitude vs. sample temperature recorded using thermal nano-analyzer instrument using hybrid AC/DC mode (**c**). Under all condition and measurement modes the analysis remained fully quantitative based on the analysis of areas under the peaks calculated for DSC heat flow (**d**), thermal nano-analyzer in DC mode (**e**) and the same in AC mode (**f**).

Having tested a range of scanning rates and measurement conditions using identical samples, we then fixed the scanning rates and compared the performance of both instruments with a range of different sample loads. Heating thermograms were generated for PEG samples ranging from milligrams to nanograms (Figure 5a,b). DSC detection limit for PEG was ~20 µg (Figure 5a) whilst for thermal nano-analyzer it was below ~30 ng. Comparison of the results from Figure 3, Figure 4 and Figure 5 demonstrate superiority of the thermal nano-analyzer over conventional DSC in terms of sensitivity achieved using fast scanning rates of which a traditional DSC is not normally capable. Our next challenge therefore was to test if slow temperature scanning rates, more suitable for use with bulk materials or life science applications, as well as being comparable to traditional DSC measurements (such as exemplified in Figure 5a), could be tolerated by a chip-based nano-analyzer. The solution allowing a slow down of the inherently fast scanning technique was achieved by adding a small AC heat modulation in addition to the controlled heating/cooling performed in DC mode (the so-called hybrid AC/DC mode). Thermograms in Figure 5b,c illustrated fast DC mode and hybrid AC/DC mode, respectively used with the same set of PEG samples. The addition of the AC readout allowed reduction of the scanning rate from 1000 °C min^−1^ to 50 °C min^−1^, comparable to the rate used in conventional DSC analysis (compare Figure 5a and Figure 5c). Modulation amplitude was recorded in AC/DC mode and plotted as the Y axis in Figure 5c instead of the heat flow or difference in temperature used in DC mode (Figure 5a,b). The endothermal peak corresponding to melting of PEG crystals is clearly visible in all three analyzes (Figure 5a–c). The sensitivity range in the hybrid mode even at slow heating rates (50 °C min^−1^) was maintained to below ~30 ng of PEG in the AC/DC mode. Under all condition and measurement modes the analysis remained fully quantitative (Figure 5d–f).

**Figure 6:**
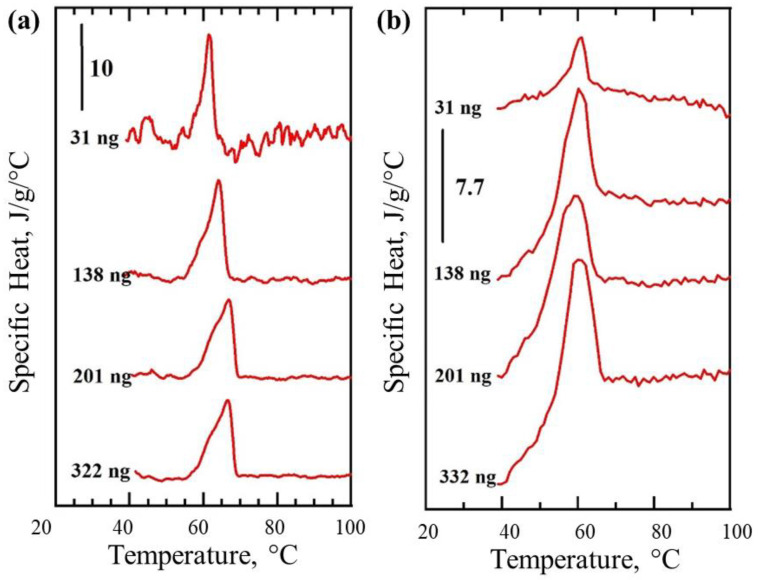
Specific heat changes during melting of polyethylene glycol (PEG) determined using thermal nano-analyzer instrument in DC and AC/DC modes. Specific heat vs. sample temperature derived from heat flow recorded in DC mode (**a**). All graphs are shown to the same scale in (**a**). Specific heat changes during PEG melting, estimated from the recorded profile of heat modulation AC amplitude. Calibration is done using PEG heat of fusion value measured in DC mode (**b**).

The DC heat flow thermogram (Figure 5b) could be converted and expressed as a function of specific heat v sample temperature (Figure 6a). Because the material (PEG) was identical in all cases, even though its amount varied, the specific heat recorded remains the same for all particles measured by our instrument in the DC mode, as expected (Figure 6a). Unlike traditional power compensating instrument designs, the AC heat component in our instrument (in the hybrid AC/DC mode) is constant and therefore under certain conditions may be assumed to not change and be a non-negative constant value. The melting of PEG (Figure 5c) satisfies these conditions, therefore, the only two remaining variables in the equation describing AC heat flow component (Equation (6)) remain the heat capacity *mc* and the measured AC temperature modulation response amplitude *T_mod_*. Therefore, under such conditions the change to the specific heat *c*, e.g., during phase transition such as melting, should be inversely proportional to the recorded *T_mod_* and the sample mass *m*.

That should allow quantitative estimates of the changes (fold difference) to the observed specific heat *c*. We therefore expressed the AC thermograms in terms of heat capacity (Figure 6b) which yielded profiles, with the melting peaks appearing nearly identical to the traditionally calculated specific heat (Figure 6a) and the areas under the peaks being proportional the *T_mod_* changes recorded (Figure 6b). This experiment proves that AC component provides an alternative way to quantify the samples or phase transitions observed. However, unlike the DC mode, the AC sensing is characterized by the limited AC penetration depth, which should allow localized thermal analysis. If the thermal penetration depth is small compared to the critical dimensions of the sample, the determined value of the heat capacity will reflect the heat capacity of the part of the sample being sensed, not the entire sample (Supplementary Figure S2).

**Figure 7:**
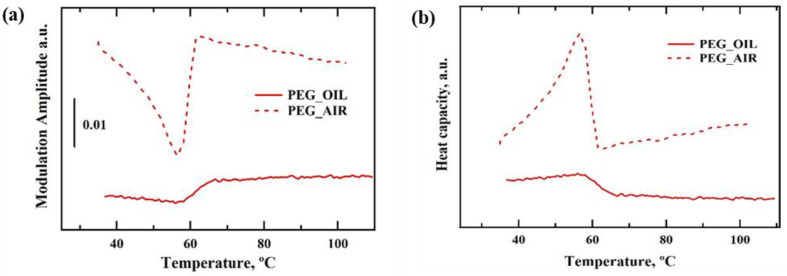
Melting of polyethylene glycol (PEG) recorded in bulk liquid using the thermal nano-analyzer instrument in hybrid AC/DC mode. Recorded heat modulation AC amplitude vs. sample temperature (**a**). Specific Heat changes estimated from the recorded profile of modulation AC amplitude (**b**). Dotted lines represent PEG in air, solid lines represent PEG measured immersed in a large volume of liquid oil.

Our final challenge was to test our setup, and the use of AC mode in particular, with a liquid cell. A customized setup with the configuration as in Figure 1b was used with a liquid cell and the detection of nanogram quantities of solid material was attempted in air and separately after immersion into bulk liquid (oil). Comparison of the temperature modulation amplitude responses during melting of the PEG particle of the same mass in air and in oil are shown in Figure 7. The mass of the PEG particle used for these measurements was ~600 ng. The modulation amplitude signal indicates polymer melting at ~57 °C, that is in line with results generated in air and reported in Figure 5 and Figure 6 for a range of different masses.

## 4. Discussion

The thermal nano-analyzer instrument reported in this work can efficiently perform the thermal analysis of a very small quantity of material both in air and if immersed in bulk liquids. The proof of principle experiments were carried out using model metal and polymer samples having a phase transition close to physiological temperature range using a commercial gas sensor in dry and liquid environments. The speed with which the heat wave is transmitted through material depends on the thermal properties of the material. The amplitude of temperature oscillation is also affected by the heat capacity of the material, which causes further attenuation of temperature oscillations. The AC penetration depth depends on thermal conductivity, specific heat capacity, sample mass and AC frequency of the heat modulation and is generally limited to the depth of heat penetration that is defined in Formula (4). The estimated mass of an individual resistive heater of the XEN-39392 sensor used can be estimated from the geometry of the heater to be 26.7 μg for the SiN_x_ membrane and 1.45 μg for the aluminum heater itself. Whilst the quoted time constant of the sensor is 4 ms [56], and assuming that the sensor behaves as a low pass filter, the amplitude of temperature oscillation is expected to gradually reduce starting from approximately 250 Hz. However, the temperature oscillations do not disappear completely and will remain detectable at much higher frequencies. Their propagation will depend on the physical characteristics of the sensor assembly, surrounding material, the sensitivity, resolution and dynamic range of the sensing circuitry. The use of thermopile sensors undoubtedly provides an advantage over resistive sensors typically used in 3*ω* sensors. Lock-in detection further reduces the noise detection threshold and the working frequency range of the system. For example, for the empty XEN-39392 sensor (no load) under ambient conditions (in air) the AC component of thermopile response remains detectable up to approximately 10,000 Hz when tested with our instrument. High-frequency AC sensing should therefore be able to detect changes in specific heat in the local vicinity of the sensor. At lower frequencies used in this work for PEG (11 Hz) the estimated AC heat penetration should be 78 μm in PEG, which ensured that the complete PEG sample was included in the AC analysis. At relatively low AC modulation frequencies (oscillation period below the time constant of the sensor), the sensors active area could therefore be assumed to be a single point heater/sensor (Figure 2). At higher frequencies that would not be the case as local temperature gradients will form [57] which will affect AC heat propagation. New calibration approaches are therefore required to achieve fully quantitative analysis at very high AC modulation frequencies. However, qualitative detection remains possible and relative changes in specific heat in the locality of AC sensors remain detectable.

The onset of indium melting temperature at 160 °C (Figure 3) was the same for both the commercial DSC instrument and thermal nano-analyzer and is in a good agreement with the literature [52]. Unlike metals polymer materials often show multiple phase transitions (e.g., glass transition, crystal to liquid crystal, isotropisation) and their structure strongly depends on the thermal history (i.e., scanning rate). In our case the model PEG sample shows a single peak corresponding to melting of polymer crystals with peak maximum at a temperature of 57 °C, in good agreement with the literature [58].

Although fast scanning rates could be advantageous for screening [59] or quality control [60] of samples or to achieve new exotic states of matter, sometimes potential users still need to able to carry out analysis at slow rates comparable with conventional DSC. For example, the open sensor design holds promise for combining the thermal nano-analyzer with other analytical methods such as IR spectroscopy or mass spectroscopy. In this case, recording time could be limited by the exposure time of the complimentary technique and the thermal analysis experiment will require minutes or even hours.

PEG melting peaks shown in thermograms obtained in DC and AC/DC mode have a maximum at 57 °C that matches well with the maximum of peak observed on the DSC thermograms (Figure 5a–c). Specific heat measurements using the AC component remained quantitative (Figure 5c), even though their derivation from *T_mod_* could only be achieved under certain conditions (Equation (6)). The areas under peaks recorded in DC mode and shown in Figure 6a are virtually identical, as expected, and represent the same heat of fusion for the polymer used (independent from the mass measured). Correction for mass was also conducted in Figure 6b, hence areas under the peaks remain proportional to the recorded heat of fusion, which should be almost identical for all the samples. Excitingly, the detection of ‘ng’ level quantity of PEG melting inside bulk liquid was possible in AC mode. Direct quantitative comparison of the data from air and oil (Figure 7) is challenging because AC-defined measurement depth and volume, when immersed in liquid, will necessarily include a small volume of that liquid and the resulting specific heat changes during PEG melting will be dampened by the presence of non-changing heat capacity of the bulk medium. Therefore, one option for optimizing such measurements might be to adjust AC frequency to limit the sensing area to the sample only. The use of very high AC will surely reduce the effect of bulk material on the sensor, but at a cost of reduced overall signal, making such detection dependent on absolute sensitivity of the sensor.

## 5. Conclusions

The AC/DC modulated thermal nano-analyzer capable of sensing nanogram quantities of material in air or in bulk liquid was built and tested. The detection principle used in our sensor utilizes localized heat waves, which under certain conditions confine the measurement area to the surface layer of the sample in the close vicinity of the sensing element. Our custom-built instrument is compatible with commercially available sensors and could be used under ambient conditions or adapted for use with a liquid cell. To illustrate the capabilities of the instrument model materials were used with detectable phase transitions at physiological temperatures and measurements were conducted in air and in oil. AC heating provides an independent way to analyze and quantify thermal properties of samples or phase transitions observed. The new instrumental setup reported in this work is suitable for measurements in bulk liquids. We do acknowledge the fact that studies using micro- and nano-sized liquid cells have been reported in the past (see for example [33,48,49]), however the possibility of being able to probe samples in bulk liquid media, rather than miniaturized liquid cells, has not been reported to the best of our knowledge. The high-frequency AC-modulated mode might potentially allow localized calorimetric measurements of biological objects in vivo.

## Data Availability

The data presented in this study are available on request from the corresponding authors.

## Supplementary Materials

The following supporting information can be downloaded at: https://www.mdpi.com/article/10.3390/nano12213799/s1, Figure S1: Thermal nano-analyzer instrument; Figure S2: Heat capacity changes recorded during melting of polyethylene glycol (PEG) determined using thermal nano-analyzer instrument in AC/DC modes with different AC modulation frequencies.
