# Supplementary material for: AC/DC Thermal Nano-Analyzer Compatible with Bulk Liquid Measurements
Source: Nanomaterials (Basel). 2022 Oct 28;12(21):3799. doi: 10.3390/nano12213799 (PMC9655476; doi:10.3390/nano12213799)
Supplement: Supplementary file 1 [file nanomaterials-12-03799-s001.zip › nanomaterials-1868390-supplementary.pdf]

Supplementary Information

AC/DC thermal nano-analyzer compatible with bulk liquid measurements

Yaroslav Odarchenko[1,*], Anna Kaźmierczak-Bałata[2], Jerzy Bodzenta[2], Enrico Ferrari[3], Mikhail Soloviev[1,*]

[1] Department of Biological Sciences, Royal Holloway University of London, Egham, Surrey TW20 0EX, UK
[2] Institute of Physics, Center for Science and Education, Silesian University of Technology, Konarskiego 22B, 44-100 Gliwice, Poland
[3] Department of Life Sciences, University of Lincoln, Lincoln LN6 7TS, UK
* Correspondence: yaro@finden.co.uk (Y.O.); mikhail.soloviev@rhul.ac.uk (M.S.)

Supplementary Informaiton

Thermal nano-analyzer input/output circuits

The fully programmable DC heating DAC board output ('slow' or 'fast' heating) is fed into the nano-analyzer heater element(s) through a unity gain isolation amplifier with high input and low output impedance. That output circuit is built using high speed, BiFET precision operational amplifiers (AD711) configured as voltage follower (with voltage gain = 1). The fully programmable DAC board output, controlling AC heating, AC modulation and AC offset, is fed into the nano-analyzer heater element(s) through a transconductance amplifier. That circuit employs a high speed, BiFET precision operational amplifier (AD711) to convert the programmed DAC voltage output into the (fully controlled/programmed) electric current for the chip resistive heaters.

The actual driving voltage generated on the resistive heater(s) is monitored independently. That voltage readout is pre-amplified with a non-inverting high speed precision operational amplifier (AD711), used as additional input buffers for the acquisition board. In addition to that, the temperature modulated component of the voltage drop generated on the resistive heater(s) is recorded separately. The signal is pre-amplified using a very low noise, low distortion, wide bandwidth and wide dynamic range operational amplifier (AD797), configured as a differential wide band DC/AC line receiver set to extract and amplify the temperature modulated component from the voltage drop generated on the resistive heater(s). That signal is further amplified using non-inverting high speed precision operational amplifier (AD711), which also serves as additional input buffer for the acquisition board.

A separate preamplifier is used to extract the thermopile sensor output signal. This pre-amplifier comprises a low noise, high accuracy instrumentation amplifier AD620A (40 ppm maximum nonlinearity, 10 GOhm, 2 pF input impedance, 800 kHz dynamic response at –3 dB Bandwidth). The AC component of that signal is then extracted from the pre-amplified thermopile output signal using a first-order high pass filter (fc=0.16Hz), and further amplified using a non-inverting high speed precision operational amplifier (AD711).

A separate non-inverting high speed precision operational amplifier (AD711) is used to pre-amplify the additional auxiliary sensor signal (from the thermocouple present on some commercial nano-calorimeter chips or from external auxiliary sensors). All such pre-amplified signals are then fed into the analogue inputs of the ADC-DAC acquisition board, where the acquisition input range can be further adjusted by the control software (automatically or manually).

The software-driven instrument allows isothermal measurements, DC heating ramps and an independent modulated AC heating (frequency range 0 to 30 kHz)), with or without DC heating. A built-in software lock-in analyzer operates within the ADC sensor frequency range (0 to 1 MHz).

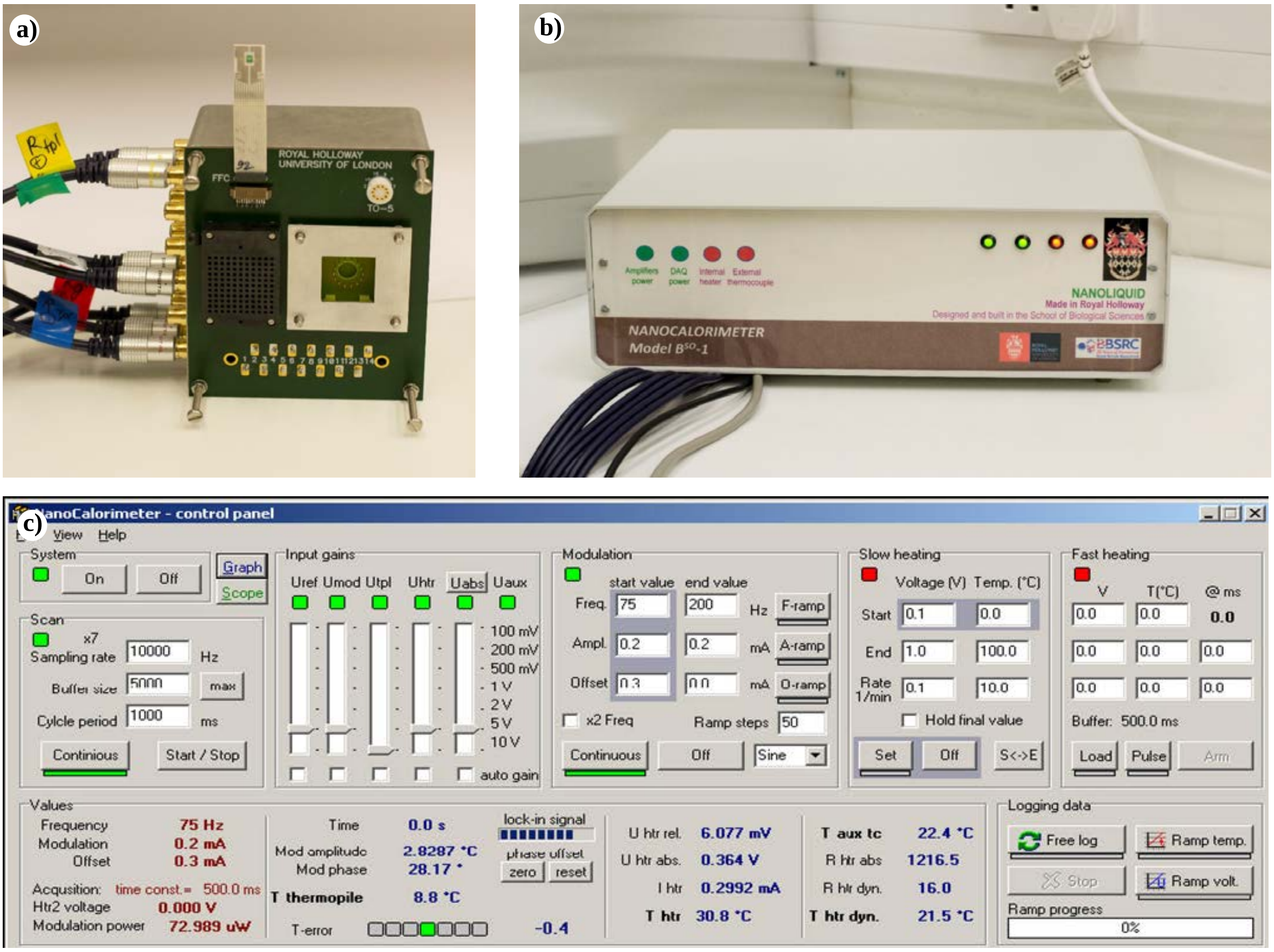

Supplementary Figure S1. Thermal nano-analyzer instrument. (a) Universal sample holder. (b) Instrument unit containing input/output circuits and the AC/DC converter. (c) Screenshot of the software control panel.

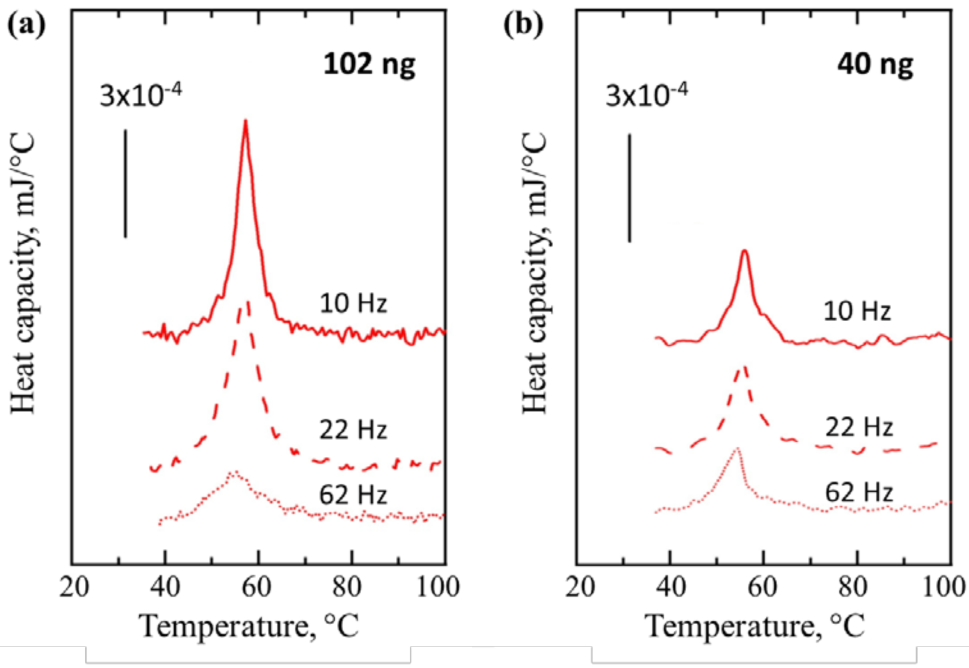

Supplementary Figure S2. Heat capacity changes recorded during melting of polyethylene glycol (PEG) determined using thermal nano-analyzer instrument in AC/DC modes with different AC modulation frequencies. Heat capacity, derived from the heat modulation AC amplitude v sample temperature, recorded for (a) 102 ng, and (b) 40 ng PEG particles using our thermal nano-analyzer instrument and hybrid AC/DC mode at the fixed scanning rate of 50 °C/min, and measured using three different AC heat modulation frequencies. The large reduction of the heat capacity recorded using higher AC heat modulation frequency with the larger particle (a) indicates that thermal penetration depth becomes smaller compared to the dimensions of the PEG sample measured. No substantial reduction is seen with the second (smaller) PEG sample (b), indicating that all three AC heating frequencies penetrate the entirety of the material in (b) but not in (a) where the higher frequency AC modulation becomes limited to the local vicinity of the sensor.
